# Diagnostic error in veterinary imaging: an underexplored field and future directions for observer performance research

**DOI:** 10.3389/fvets.2026.1978054

**Published:** 2026-10-21

**Authors:** Silvia Burti, Alessandro Zotti, Simon Coghlan, Tommaso Banzato

**Affiliations:** 1 Department of Animal Medicine, Production and Health, University of Padua, Legnaro, Italy; 2 School of Computing and Information Systems, Centre for AI and Digital Ethics, Australian Research Council Centre of Excellence for Automated Decision-Making and Society, University of Melbourne, Melbourne, VIC, Australia

**Keywords:** diagnostic error, human factors, observer performance, radiology, veterinary imaging

## Abstract

Diagnostic error is increasingly recognized as an important component of diagnostic quality in human radiology, where decades of research have established observer-performance as a measurable scientific process shaped by perceptual, cognitive, organizational, cultural, and technological factors. In veterinary diagnostic imaging, however, diagnostic error remains comparatively under-investigated, and existing evidence is fragmented across studies of diagnostic accuracy, observer variability, image quality, and quality assurance. In this perspective, we examine how concepts developed in human radiology may inform the study of diagnostic error in veterinary imaging, while considering the distinctive clinical, methodological, organizational, and ethical characteristics of veterinary practice. We argue that veterinary diagnostic imaging would benefit from recognizing diagnostic error as a distinct field of scientific investigation and from developing observer-performance research as a systematic area of investigation. We identify four interconnected priorities: developing robust methods to measure diagnostic performance, investigating the mechanisms underlying diagnostic error, evaluating interventions to improve observer performance, and building collaborative and multidisciplinary research networks. Together, these priorities provide a basis for moving from the recognition of diagnostic discrepancies toward a systematic science of diagnostic error aimed at improving diagnostic performance, quality assurance, education, and ultimately patient care and welfare.

## 1 Introduction

Diagnostic imaging plays a central role in contemporary veterinary medicine, supporting clinical decision-making across a wide range of species, clinical settings, and disease processes. Radiography, ultrasonography, computed tomography (CT), and magnetic resonance imaging (MRI) have progressively expanded the diagnostic capabilities of both specialist radiologists and general practitioners, increasing both the complexity and the interpretative demands of imaging practice. The human radiology literature suggests that interpretive discrepancy rates remain substantial despite major advances in imaging technology, indicating that technological progress alone may be insufficient to eliminate variability in image interpretation. Over time, human medicine has developed a structured “science of diagnostic error,” integrating observer-performance studies, discrepancy analysis, human factors research, quality assurance systems, and educational strategies aimed at understanding and mitigating interpretative error (1). These concepts have profoundly influenced modern radiology and now constitute an established field of research.

Yet compared with human radiology, veterinary diagnostic imaging has a less consolidated and more fragmented body of knowledge dedicated to the systematic study of diagnostic error. Existing veterinary literature has primarily focused on diagnostic accuracy, radiographic technical quality, inter-observer variability, technical error, and emerging quality-assurance initiatives (2–8). However, only one recent study has directly quantified radiologic error through systematic postmortem audit, distinguishing true radiologic errors from non-error discrepancies (9). Despite this important contribution, diagnostic error remains poorly investigated in veterinary diagnostic imaging (10). Consequently, veterinary imaging still lacks an integrated observer-performance perspective comparable to that developed in human radiology. Additional challenges—including species variability, non-verbal patients, heterogeneous clinical settings, and the increasing reliance on teleradiology—may further complicate the recognition and systematic investigation of diagnostic error in veterinary imaging.

Understanding and addressing diagnostic error is an important ethical responsibility of the veterinary profession. The profession aims to improve animal health and wellbeing for the sake of the animals and the humans who depend on them (11, 12). Professional codes of conduct enjoin veterinarians to “ensure the health and welfare of animals committed to their care” by exercising and maintaining “professional competence” (13). Competence clearly involves the goal of eliminating or reducing diagnostic errors that could harm animal patients. Unfortunately, harmful errors are common in veterinary medicine (14, 15), just as they are in human medicine (16). Given that the capabilities and role of imaging modalities in veterinary medicine continue to expand – and that other technological advances such as artificial intelligence (AI) may introduce new and still poorly understood sources of diagnostic error – the need to better understand and tackle such error is urgent.

To address the above gaps in understanding, we first revisit how, in human radiology, understanding of diagnostic error evolved from focusing on individual failing into a structured field of investigation. We use the term “*diagnostic error research*” to refer to the broader effort to understand, prevent, and learn from diagnostic error; and the term “*observer performance research*” to refer more specifically to the investigation of the perceptual and cognitive determinants of diagnostic imaging interpretation and of factors influencing observer performance. Because diagnostic imaging fundamentally relies on human observers, observer-performance research has become one of the principal scientific approaches through which diagnostic error has been investigated in radiology. We then examine whether these mechanisms have begun to be investigated in veterinary diagnostic imaging and, finally, identify future directions for developing a more integrated observer-performance evidence base.

## 2 Diagnostic error in human radiology: from individual failure to observer-performance research

### 2.1 From discrepancy to observer-performance research

The recognition of diagnostic error in human radiology emerged progressively throughout the second half of the twentieth century. Early work by Garland starting in the late 1940s represented one of the first major acknowledgments that radiologic interpretation was intrinsically vulnerable to observer error, challenging the prevailing assumption that diagnostic discrepancies primarily reflected insufficient knowledge, negligence, or inadequate imaging quality (17–19). Subsequent reviews later described these observations as having profoundly influenced the radiologic community by establishing that even experienced radiologists could fail to identify visible abnormalities on imaging studies (18, 20, 21).

For several decades, radiologic error was approached predominantly as a retrospective discrepancy problem, largely quantified through disagreement between original reports and subsequent review. Importantly, discrepancy and diagnostic error are related but distinct concepts. A discrepancy indicates disagreement between interpretations or reports, whereas a diagnostic error implies that an interpretation is incorrect according to an appropriate reference standard. Thus, not all discrepancies represent diagnostic errors (9). Early literature focused primarily on estimating discrepancy prevalence and categorizing missed findings, while the underlying mechanisms of interpretative error remained less clearly defined. During this period, multiple studies suggested that overall radiologic discrepancy rates remained relatively stable despite major advances in imaging technology, indicating that technological progress alone was insufficient to eliminate variability in image interpretation (16, 21, 22). The major conceptual milestones underlying this evolution are summarized in Figure 1.

**Figure 1:**
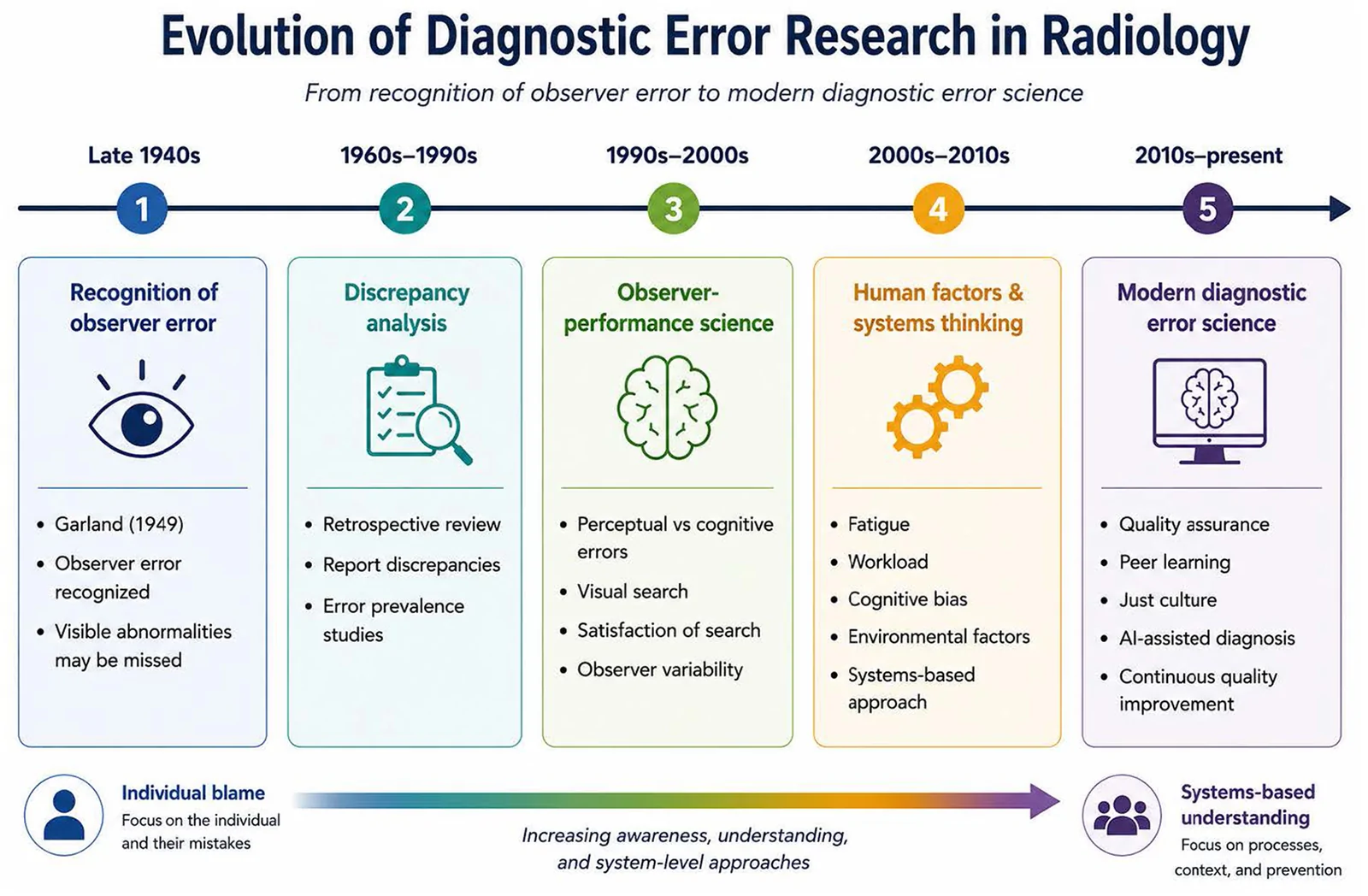
Evolution of research on diagnostic error in radiology.

Over time, however, research in radiology demonstrated that diagnostic errors are not isolated anomalies, but recurrent and partially predictable phenomena arising from complex interactions between human cognition, perceptual limitations, environmental conditions, workflow organization, and broader system-level factors (23, 24). By the late twentieth and early twenty-first century, radiology research shifted from simple discrepancy counting toward a more structured observer-performance framework. One of the most important conceptual developments was the distinction between perceptual and cognitive errors. Perceptual errors occur when an abnormality visible on the image is not detected by the observer, whereas cognitive errors involve incorrect interpretation of a detected finding (1, 16, 18, 25–27). This distinction profoundly changed the understanding of radiologic performance by shifting attention from purely interpretative competence toward the mechanisms governing visual search, perception, and diagnostic reasoning.

Subsequent observer-performance research further demonstrated that diagnostic interpretation is strongly influenced by cognitive biases and contextual factors. Phenomena such as satisfaction of search, anchoring bias, inattentional blindness, and framing effects have all been shown to alter radiologic decision-making (24, 28). Satisfaction of search, for example, describes the tendency to prematurely reduce the intensity of image evaluation after identifying an initial abnormality, thereby increasing the likelihood of missing additional findings (24). These cognitive and perceptual phenomena are briefly summarized in Table 1.

**Table 1:** Selected cognitive and perceptual phenomena influencing diagnostic interpretation in radiology.

Phenomenon	Brief description	References
Anchoring bias	Reliance on an initial diagnostic impression without sufficient adjustment in light of subsequent information	(24)
Framing bias (framing effect)	Tendency for diagnostic interpretation to be influenced by how the clinical problem or contextual information is worded or presented	(24, 62)
Satisfaction of search	Reduced likelihood of detecting additional abnormalities after an initial finding has been identified	(24, 28)
Inattentional blindness	Failure to notice an unexpected but visible abnormality because it falls outside the observer's current attentional set or usual search pattern	(24)

Human radiology increasingly incorporated concepts derived from human-factors science, cognitive psychology, and systems-based patient-safety models. Diagnostic performance came to be understood as highly sensitive to fatigue, workload, interruptions, environmental stressors, time pressure, case complexity, and cognitive overload. Multiple studies demonstrated measurable declines in detection performance associated with prolonged reading sessions and high-volume reporting (29–32). These findings transformed radiologic discrepancy from a purely retrospective quality metric into a measurable and scientifically investigable component of observer performance (16).

The emergence of observer-performance science represented a further step in this evolution. Radiology developed dedicated methodologies to study how radiologists search, perceive, interpret, and report imaging findings, while complementary human-factors approaches sought to analyse diagnostic errors across the entire imaging workflow rather than focusing exclusively on image interpretation (16, 19, 33–36). Observer-performance studies, eye-tracking analyses, search-pattern research, double-reading protocols, workload studies, and secondary-interpretation analyses all contributed to the understanding that diagnostic interpretation is itself a measurable scientific process (16, 19, 34–36). Within this framework, variability between observers is no longer viewed solely as a limitation, but also as a valuable source of information regarding the cognitive and perceptual mechanisms underlying diagnostic practice (20, 27). More recently, AI and computer-assisted detection systems are increasingly being explored as potential support tools within radiology workflows. Contemporary radiology therefore approaches diagnostic performance as the result of dynamic interactions between observer cognition, imaging technology, organizational systems, and increasingly, advanced computational support systems (18, 21). Taken together, these developments established observer-performance research as a mature conceptual framework for studying diagnostic performance in radiology.

### 2.2 From blame to learning: the emergence of just culture

This conceptual evolution also transformed the management of diagnostic error within radiology departments. Many institutions progressively implemented structured discrepancy-review systems, peer-review programs, quality-assurance initiatives, and discrepancy conferences aimed not primarily at punishment, but at reflective learning and performance improvement (37–39). Communication-related factors also became increasingly recognized as potential contributors to delayed or missed diagnoses, further expanding the understanding of diagnostic error beyond image perception alone (21, 27).

Importantly, this transition paralleled the development of the “just culture” model in healthcare (40–42), which draws on the extensive understanding of how errors and mistakes occur in other high stakes fields like engineering and aviation. The just culture model emphasizes analysis of underlying mechanisms contributing to error rather than applying punitive responses and blame (43, 44). This shift from localized blame to impartial analysis of various causes that enter at multiple levels (45) occurred for several reasons. First, even the most expert and competent individuals can make errors that are not highly culpable. Blaming and shaming can sometimes be unfair and can create a “second victim” in the practitioner who pays a psychological or professional penalty. Second, punitive reactions that create fear can prevent error disclosures that may help prevent future errors. Third, errors can be made because of many causal factors beyond the individual, which again must be identified to improve safety. For example, in veterinary medicine as in human medicine, high workload, burnout, lack of support, and mental illness (46) can affect the performance of otherwise competent practitioners.

Adopting a just culture approach may therefore be required in professions with duties to improve patient health and wellbeing. As such, the model can be regarded as a means of ensuring proper accountability not only in human medicine, but also in veterinary medicine (14). It is important for the veterinary profession to correctly understand the just culture model. For a start, it is not a blame-free approach that dismisses punitive responses altogether: misdiagnoses that are negligent or grossly incompetent, and that risk serious harm to patients, may still be properly sanctioned. Nevertheless, the just culture model also recognizes that many harms result from errors that are not blameworthy and are not best addressed through administering penalties.

Just culture recognizes several classifications of behavior causing risk or harm (41). *Recklessness* involves a conscious disregard of risky behavior which may call for punitive responses. *At-risk behavior* involves a failure to recognize risk or else to mistakenly think it justified; an appropriate response may be to coach these practitioners. *Human error* involves mistakes that create risk or harm but for which punitive action may be both unfair to the doctor and unhelpful for present and future patients. Nonetheless, human error can be recurrent and caused or exacerbated by a range of factors that can potentially be identified and addressed (41). The maturation of healthcare professions involves recognizing these behavioral classifications without confusing them, understanding their root causes using scientific means, and finding the most effective and fair ways to address them.

## 3 Diagnostic error research in veterinary diagnostic imaging

Veterinary studies have previously investigated radiographic interpretation errors and factors associated with observer performance, particularly in educational settings (47), while more recent work has directly examined radiologic errors and their perceptual and cognitive contributors in clinical practice (9). However, systematic investigation of these phenomena within a broader observer-performance framework remains limited.

One potentially important characteristic of veterinary diagnostic imaging concerns the availability and quality of clinical information. In veterinary medicine, clinical history and reported symptoms are necessarily mediated through animal owners and referring veterinarians. Veterinary radiologists may receive clinical histories that are incomplete or heterogeneous and that necessarily reflect, to some extent, the owner's observations and the referring clinician's interpretation. The importance of contextual information has already been demonstrated in canine thoracic radiography, where access to pertinent clinical information and signalment significantly improved diagnostic accuracy (48). Further research is needed to determine how the availability, quality, and source of such information influence diagnostic interpretation across different imaging modalities and clinical settings.

Another major challenge relates to the establishment of reliable reference standards. In veterinary diagnostic imaging, radiologists often contribute at a specific stage of the diagnostic pathway and may not have access to subsequent clinical outcomes or confirmatory testing. Depending on the disease and clinical context, appropriate reference standards may include histopathology or cytology, surgical findings, laboratory or microbiological testing, longitudinal clinical follow-up, repeated or alternative imaging, or combinations of these approaches. However, such information may not always be obtained and, even when available, may not necessarily be accessible to the interpreting radiologist. Consequently, both the availability and accessibility of reference-standard information may complicate the assessment of diagnostic performance and limit opportunities for feedback and learning (48–50).

Recent veterinary literature has begun to describe diagnostic error as a multifactorial phenomenon arising from perceptual and cognitive limitations, methodological and technological constraints, and broader organizational factors rather than from individual failure alone (51). Although this evidence remains fragmented, several components of diagnostic error research have already begun to emerge within the veterinary imaging literature.

Studies have demonstrated substantial observer variability while also highlighting the importance of structured image-quality criteria and standardized assessment protocols for ensuring high diagnostic quality (2, 52). Experimental and educational studies have also begun to explore the perceptual and cognitive aspects of diagnostic performance, including satisfaction of search, visual observation skills, and diagnostic reasoning (53–55). Experience has also been associated with lower observer variability and fewer major diagnostic discrepancies, suggesting that diagnostic performance may improve through training, although the educational pathways underlying this process remain poorly understood (3). Importantly, image interpretation in veterinary practice is not restricted to specialist radiologists. Particularly in first-opinion practice, radiographic examinations may be interpreted by general practitioners with varying levels of training and experience in diagnostic imaging (3, 8).

Veterinary diagnostic imaging is also characterized by considerable organizational heterogeneity. Imaging examinations are interpreted in various clinical settings, including first-opinion practice, referral hospitals, academic institutions, and commercial teleradiology services, the latter of which has expanded rapidly in the past decade (8). These settings differ substantially in case complexity, workload, access to clinical information, and level of subspecialisation, while deficiencies in image acquisition, transmission, documentation, or quality control may directly affect the standard of patient care (8, 56). Although second-reader agreement may provide a useful quality-control measure, concordance between readers does not necessarily establish diagnostic correctness, as both observers may share the same incorrect interpretation. Because diagnostic error may also arise from systemic and organizational pressures rather than individual failure alone (51), these differences represent plausible determinants of diagnostic performance, but remain largely unexplored.

Although still fragmented, the available evidence suggests that veterinary diagnostic imaging is beginning to address many of the same questions that led to diagnostic error research in human radiology. However, systematic investigation of these questions within an integrated observer-performance perspective remains limited, providing the rationale for the research priorities proposed in the following section.

## 4 Future directions for observer-performance research in veterinary diagnostic imaging

Building on this evidence, future research should move beyond documenting discrepancies to investigate the mechanisms underlying diagnostic performance and translate this knowledge into measurable improvements in clinical practice. While several of the principles underpinning observer-performance research are well-established in human radiology, their investigation in veterinary diagnostic imaging requires consideration of the specific clinical, methodological, and organizational characteristics outlined above. In our view, four interconnected priorities may provide a framework for future research, as summarized in Figure 2.

**Figure 2:**
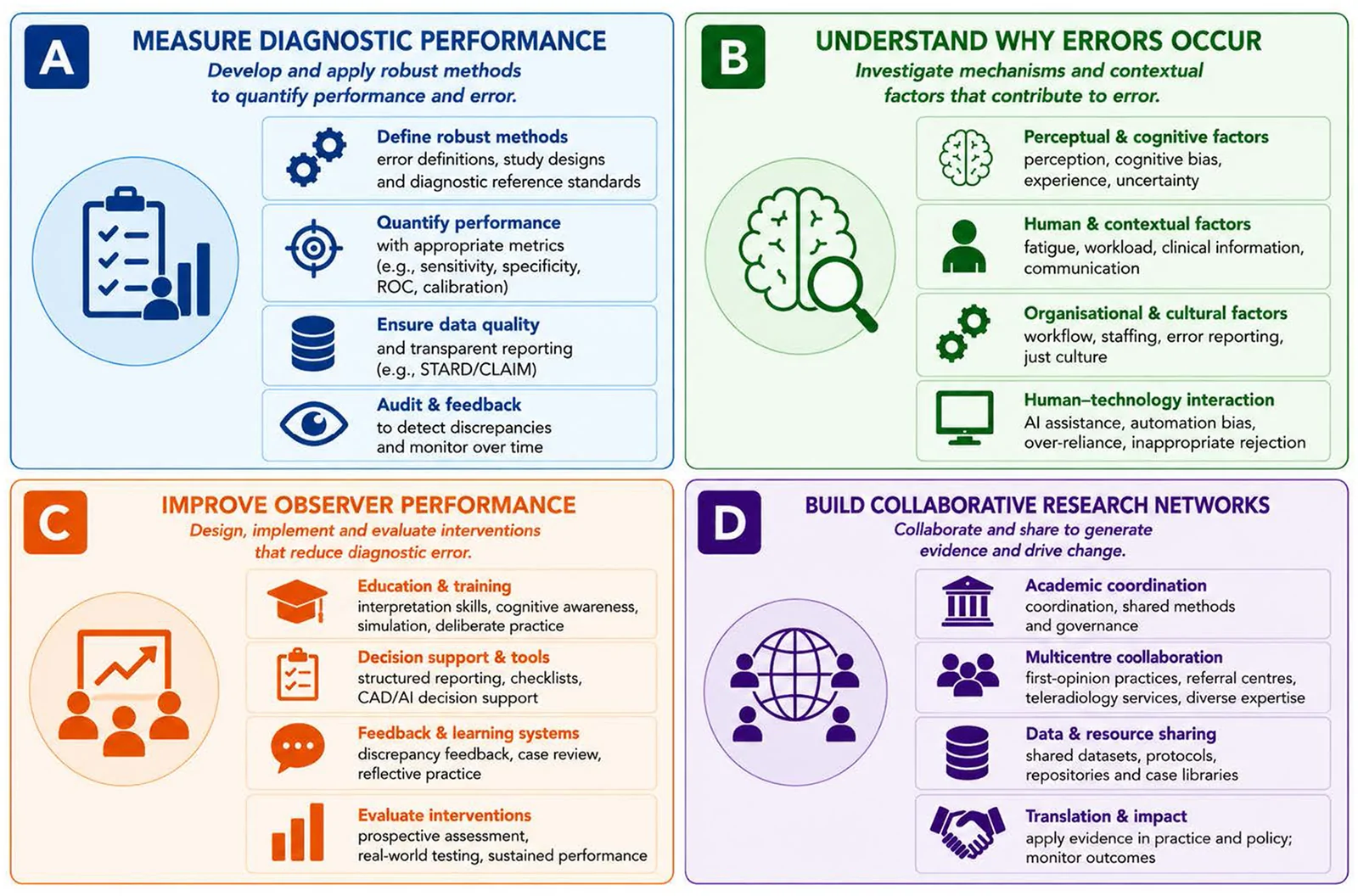
Four interconnected priorities for advancing observer-performance research in veterinary diagnostic imaging: **(A)** measure diagnostic performance; **(B)** understand why errors occur; **(C)** improve observer performance; and **(D)** build collaborative research networks.

### 4.1 Priority 1—Measure diagnostic performance

The first priority is the development of robust and reproducible methodologies for measuring diagnostic performance. Reliable disease-appropriate diagnostic reference standards, standardized definitions of diagnostic error, and well-designed observer-performance studies are essential for generating robust evidence that is comparable across institutions and imaging modalities. Consensus or double-reading protocols may help identify interpretive disagreement, but should not be considered substitutes for a diagnostic reference standard when diagnostic correctness is being assessed. Future studies should also adopt consistent performance metrics and transparent reporting standards, allowing results to be compared across studies and eventually supporting the development of meaningful benchmarks for veterinary diagnostic imaging. At the institutional level, structured discrepancy review using predefined definitions and consistent recording criteria could provide a practical starting point for monitoring diagnostic performance over time. These core methodological foundations, summarized in Figure 2A, will allow veterinary imaging to move beyond isolated discrepancy reports toward systematic performance assessment.

### 4.2 Priority 2—Understand why errors occur

Once diagnostic performance can be measured consistently, research should focus on understanding the mechanisms underlying diagnostic error. Rather than considering discrepancies as isolated outcomes, future studies should investigate the relative contributions of perceptual processes, cognitive biases, experience, fatigue, workload, workflow organization, and environmental factors. Multicentre observer-performance studies could examine how the contribution of these factors varies across veterinary practice settings, including first-opinion practices, referral centers, and teleradiology services. As AI-based decision-support systems become increasingly capable and prevalent, research on diagnostic error should also examine their interaction with human observers. As a distinctive technology that performs perceptual and cognitive activities previously reserved for humans (57), AI has the potential to both assist medical diagnosis and increase error through human factors such as automation bias and over-reliance (58, 59). Another source of error could occur when practitioners inappropriately ignore reliable AI recommendations (60). Observer studies comparing unaided and AI-assisted interpretation could help determine when AI improves diagnostic performance and when it introduces new sources of error. Addressing these questions may require collaboration between veterinarians, AI specialists, and human-computer interaction researchers.

Another possible focus concerns whether lessons from just culture in human medicine could be adapted to veterinary practice. Rather than promoting either a punitive blame culture or a permissive approach that downplays professional accountability, just culture seeks to balance accountability with patient safety and learning from errors. Research could examine how different approaches to discrepancy reporting, feedback, and error discussion affect practitioner willingness to report errors, learning, wellbeing, and diagnostic performance in veterinary settings (Figure 2B).

### 4.3 Priority 3—Improve observer performance

The next step is to translate this knowledge into practical interventions. Educational programmes, structured feedback, discrepancy-review meetings, quality-assurance initiatives, and decision-support technologies should be evaluated not only for their educational value but also for their ability to improve diagnostic performance in everyday clinical practice. At the institutional level, structured feedback, regular discrepancy-review meetings, and targeted educational interventions represent feasible measures that veterinary hospitals and clinics could implement and prospectively evaluate. For example, while AI poses risks of misdiagnosis (61), it could also serve as a decision-support tool to minimize observer error by identifying areas of radiographic interest or querying observer judgements (60). Effective human oversight also requires sufficient domain knowledge to critically evaluate AI-generated outputs. AI should therefore complement rather than replace diagnostic expertise, as observers who lack the knowledge required to independently assess a finding may also be unable to recognize an incorrect AI recommendation. Observer-performance research should therefore evolve from descriptive studies toward interventional studies that test whether such measures produce measurable improvements in diagnostic performance. Importantly, these interventions should ultimately be evaluated under real-world clinical conditions to determine whether the improvements observed in controlled settings translate into sustained benefits in routine practice (Figure 2C).

### 4.4 Priority 4—Build collaborative research networks

Achieving these objectives will require collaboration across veterinary institutions, referral centers, first-opinion practices, and teleradiology services. Multicentre observer-performance studies involving diverse clinical environments, imaging modalities, and levels of observer experience will be important for producing robust and generalisable evidence. Collaborative research networks will also facilitate the development of shared protocols for recording and classifying discrepancies and diagnostic errors, common methodologies and performance metrics, benchmarking initiatives, and large datasets for future AI applications. Multidisciplinary collaboration should also extend beyond diagnostic imaging to include expertise from fields such as social sciences, veterinary ethics, veterinary education, human factors, and human-computer interaction. This collaboration will be particularly important for investigating aspects of diagnostic performance that cannot be adequately addressed through imaging research alone, including cognitive, organizational, ethical, and technological factors. Academic institutions may be particularly well-positioned to coordinate these efforts, acting as hubs that integrate data and expertise from first-opinion practices, referral centers, and teleradiology services while facilitating data harmonization and comparative analysis across different clinical settings. We therefore propose that academic institutions take a leading role in establishing and coordinating multicentre diagnostic-error research networks, with active participation from the full spectrum of veterinary imaging practice (Figure 2D).

Collectively, these priorities provide a framework for developing observer-performance research that is adapted to the specific characteristics of veterinary diagnostic imaging and capable of translating evidence into improvements in education, quality assurance, and clinical practice.

## 5 Conclusions

Diagnostic error is an unavoidable challenge in diagnostic imaging, but it should not remain an unexamined one. Human radiology has shown that observer performance can be studied systematically, revealing the perceptual, cognitive, organizational, cultural, and technological factors that shape image interpretation. Veterinary imaging now has the opportunity to build on this knowledge and develop an evidence base adapted to the specific realities of veterinary practice. Recognizing diagnostic error as a legitimate field of scientific investigation is the first step toward understanding it, learning from it, and developing collaborative strategies to improve diagnostic performance, patient care, and wellbeing.

## Data Availability

The original contributions presented in the study are included in the article/supplementary material, further inquiries can be directed to the corresponding author.
